# Household Hints and Recipes

**Published:** 1884-04

**Authors:** 


### Household Hints & Recipes.

TO CLEAN IVORY.—Rub it with bicarbonate of sodium, applied by means of a tooth-brush dipped in warm water.—*Chem. and Drug.*

IRON RUST SPOT REMOVER—

Cream of tartar, 50 parts.
Binoxalate of potassium, powd., 50 parts.
Oil of rosemary, 1 part.

TO FASTEN LABELS ON METAL.—The following composition is recommended:

Mucilage of tragacanth, 10 parts.
Honey, 10 parts.
Flour, 1 part.

PESTLE HANDLES.—Mr. John M. Harvy, of Wilmington, Del., writes us that he has found a cement of litharge and glycerine, as suggested for other purposes, to work well as a fastening for pestle handles.

RATS AND MICE.—Sekerka recommends to pour common tar into the rat or mice holes, which either kills them at once, or will, at least, make the locality uninhabitable and unpassable for them, since it takes a long time to dry.

LUMBAGO may be quickly relieved by binding a piece of enameled cloth, such as is used to cover tables, over the loins outside of the flannel shirt. Profuse perspiration is produced, which rapidly relieves the pain.—*Sci. Amer.*

WATERPROOF LEATHER.—Huleux and Dreyfus recommend the following application for leather and paper:

Pure yellow wax, two pounds; Burgundy pitch, two ounces; peanut oil, two and one-half ounces; copperas, one and one-half ounces; oil of red thyme, five drachms. Melt together, and apply while warm.

TOOTHACHE.—A correspondent of the London *Electrician* gives the following as an instant remedy for toothache: With a small piece of zinc and a bit of silver (any silver coin will do) the zinc placed on one side of the afflicted gum, and the silver on the other, by bringing the edges together, the small current of electricity generated immediately and painlessly stops the toothache.

CORN, WART AND BUNION.—Mix sixteen fluid ounces of collodion with two ounces (avoir.) salicylic acid, and, when this is dissolved, add one ounce (avoir.) of chloride of zinc. Keep it tightly stoppered and away from lights or fire.

TOOTH SOAP.—Castile soap, one pound; pumice stone in fine powder, one ounce; thymol, twenty grains; oil of wintergreen, thirty drops. Shave the soap into ribbons; beat it into a paste with a little water, and add first the pumice stone, and lastly the thymol and oil of wintergreen dissolved in a small quantity of alcohol.

INDELIBLE PENCILS.—

Kaolin, 8 parts.
Finely powdered manganese dioxide, 2 parts.
Silver nitrate, 3 parts.

Mix and knead intimately with distilled water, 5 parts. Then dry the mass and enclose it in wood pencils.

CEMENT FOR GLASS.—Waechter describes the following method of preparing a white enamel for joining milk glass:

Melt together three parts of red lead, two of white sand, and three of crystallized boracic acid in a Hessian crucible. The melted mass is poured out on a plate of metal and finely pulverized. This is mixed with gum tragacanth and applied to the glass, and the pieces pressed together. Finally, it is heated in a muffle, but not enough to melt the enamel, but only to soften it enough to make it unite with the glass.

INSECTICIDE FOR PLANTS.—

Soft soap, 4 parts.
Tobacco, 6 parts.
Fusel oil, 5 parts.
Methylic alcohol, 20 parts.
Water sufficient to make 1,000 parts.

Boil the tobacco with an equal weight of water for half an hour, replacing the water as it is dissipated on boiling. Then strain. Mix this liquid extract with the other ingredients, and add enough to make 1,000 parts. Before use, the mixture is well shaken up, and then applied by means of a syringe throwing a fine spray to the affected plants.—After *The Chem. Journal.*

A rather dilute infusion of tobacco alone is likewise very effective for this purpose.

WARTS.—A plaster of black soap, applied each night for a fortnight, according to M. Vidal, will soften a wart so that it may be scraped off. The treatment by M. Cellier is to transfix the principal wart with the point of a pin, the head of which is then to be held in the flame of a candle until the wart is destroyed; it will drop off in a few days. The remaining warts will then usually disappear.—*La France Medicale.*

DANDRUFF WASH.—*Ad. Vomaca* recommends the following wash for dandruff: Borax, fifteen parts; Glycerine, thirty parts; Decoction of soap bark root, fifty parts; made up with water to 300 parts. In the morning rub the hair with a pomatum of: Tannin, two parts; tincture of cantharides, five parts; vaseline, fifty parts; balsam of Peru, five parts; oil of mace, two parts. The latest ingredient, the odor of which is objectionable to many persons but is an excellent preventive against the falling out of the hair, can be mixed with vaseline, whereby its pungent odor is disguised.

LAVING FLUID.—Take 100 parts of the finest ninety-five per cent. alcohol, and add three parts oil of neroli, 180 parts extract of patchouli, 180 parts extract of jasmin, thirty parts Peruvian balsam, fifteen parts Tolu balsam, thirty parts benzoic resin, and 400 parts water of orange flowers. The resins are first to be dissolved completely in alcohol, frequently shaking and digesting, filtered, and the other ingredients are then added. To each ten parts of the ready essence 1,000 parts of water are to be added, and with it wash the face, without using soap. Employ a cloth for washing.

PRESERVING FLOWERS, LEAVES, AND OTHER PARTS OF PLANTS.—German patent No. 23,-792, issued to J. L. Wensel in Berlin, covers the following process: The respective parts of the plant are coated with a solution of shellac or resin, until they are perfectly dry. The moisture completely escapes after some time, through the fine fissures of the coating. Finally, the coating is removed by the cautious use of alcohol, whereupon the organs will present their original sharp outline.

If it is desired to give them a better appearance, they may be coated with a thin layer of an adhesive varnish and dusted with a suitably colored metallic bronze powder.

BLEACH SPONGES.—The following is, we are informed, the method adopted in the New York Hospital: The sponges are first prepared by beating out the sand, shells, etc., and washed in warm (not hot) water. Next, they are placed for twenty four hours in a solution of permanganate of potassa made of one part of the salt to one thousand parts of water. Then they are washed with warm water, and bleached by immersion in a mixture composed of a one per cent. solution of sulphite of soda and one-fifth of its weight of an eight per cent. solution of muriatic or oxalic acid. They are left a few minutes only in the bleaching liquid, as a longer immersion renders them brittle. After being washed in an abundance of water, the sponges are placed in a five per cent. solution of carbolic acid, or a one per mille solution of mercuric chloride, where they remain till wanted for use. This process is, of course, meant for fine surgical sponges. A coarser article, intended for other purposes, would require somewhat stronger solutions, and should be dried immediately after the final washing.

VIENNA BREAD.—Bread-making is hardly to be considered germane to pharmacy, but it so happens that we have directions for making this variety of bread in the form of small loaves, which may answer your purpose. "Wiener Kaisersemmel" are made as follows:

Finest wheat-flour, 8 pounds.

Milk, 3½ quarts.

Water, 3½ quarts.

Compressed yeast, 3 1-3 ounces.

Salt, 1 ounce.

After all the materials have acquired the temperature of the baking-room, the flour is poured, in a loose heap, in the middle of the baking trough, and a small quantity of the heap, on one side, mixed to a thin dough with the milk and water previously poured together and mixed with the yeast and salt. The dough is allowed to stand three-quarters of an hour, well covered. After this time, or as soon as fermentation has begun, the dough is mixed intimately with the remainder of the flour and the rest of the liquid, and left to rise for 2½ hours. It is then cut into pieces weighing each one pound, each of which is divided into 12 square pieces of equal weight. The corners of each of these squares having been turned over to the centre, the cakes are put in the oven and baked for fifteen minutes. The heating must be uniform; if the oven is hotter in one place than in another, the cakes must be shifted about.

To impart a gloss to the cakes, they are brushed over with a sponge dipped in milk.

